# Maternal preterm birth prediction in the United States: a case-control database study

**DOI:** 10.1186/s12887-022-03591-w

**Published:** 2022-09-14

**Authors:** Yan Li, Xiaoyu Fu, Xinmeng Guo, Huili Liang, Dongru Cao, Junmei Shi

**Affiliations:** 1 Department of Obstetrics and Gynecology, Beijing Haidian Maternal and Child Healthcare Hospital, NO.33 Haidian South Road, Haidian District, Beijing, 100080 China; 2 Department of Obstetrics and Gynecology, the Seventh Medical Centre, Chinese PLA General Hospital, Beijing, 100853 China; 3 Department of Obstetrics and Gynecology, College of Medicine, Nankai University, Tianjin, 300071 China

**Keywords:** Preterm birth, Prediction, Nomogram, Area under the curve

## Abstract

**Background:**

Preterm birth is serious public health worldwide, and early prediction of preterm birth in pregnant women may provide assistance for timely intervention and reduction of preterm birth. This study aimed to develop a preterm birth prediction model that is readily available and convenient for clinical application.

**Methods:**

Data used in this case-control study were extracted from the National Vital Statistics System (NVSS) database between 2018 and 2019. Univariate and multivariate logistic regression analyses were utilized to find factors associated with preterm birth. Odds ratio (OR) and 95% confidence interval (CI) were used as effect measures. The area under the curve (AUC), accuracy, sensitivity, and specificity were utilized as model performance evaluation metrics.

**Results:**

Data from 3,006,989 pregnant women in 2019 and 3,039,922 pregnant women in 2018 were used for the model establishment and external validation, respectively. Of these 3,006,989 pregnant women, 324,700 (10.8%) had a preterm birth. Higher education level of pregnant women [bachelor (OR = 0.82; 95%CI, 0.81–0.84); master or above (OR = 0.82; 95%CI, 0.81–0.83)], pre-pregnancy overweight (OR = 0.96; 95%CI, 0.95–0.98) and obesity (OR = 0.94; 95%CI, 0.93–0.96), and prenatal care (OR = 0.48; 95%CI, 0.47–0.50) were associated with a reduced risk of preterm birth, while age ≥ 35 years (OR = 1.27; 95%CI, 1.26–1.29), black race (OR = 1.26; 95%CI, 1.23–1.29), pre-pregnancy underweight (OR = 1.26; 95%CI, 1.22–1.30), pregnancy smoking (OR = 1.27; 95%CI, 1.24–1.30), pre-pregnancy diabetes (OR = 2.08; 95%CI, 1.99–2.16), pre-pregnancy hypertension (OR = 2.22; 95%CI, 2.16–2.29), previous preterm birth (OR = 2.95; 95%CI, 2.88–3.01), and plurality (OR = 12.99; 95%CI, 12.73–13.24) were related to an increased risk of preterm birth. The AUC and accuracy of the prediction model in the testing set were 0.688 (95%CI, 0.686–0.689) and 0.762 (95%CI, 0.762–0.763), respectively. In addition, a nomogram based on information on pregnant women and their spouses was established to predict the risk of preterm birth in pregnant women.

**Conclusions:**

The nomogram for predicting the risk of preterm birth in pregnant women had a good performance and the relevant predictors are readily available clinically, which may provide a simple tool for the prediction of preterm birth.

**Supplementary Information:**

The online version contains supplementary material available at 10.1186/s12887-022-03591-w.

## Background

Preterm birth, defined as delivery of less than 37 completed gestational weeks [1, 2], may lead to high rates of maternal and perinatal morbidity and mortality worldwide. The global rate of preterm birth is approximately 11%, affecting 15 million newborns every year, and preterm birth is the leading cause of child mortality, accounting for 35% of all child deaths [3]. In the United State, the preterm delivery rate due to insufficient gestation age is approximately 9–10% [4]. The annual economic burden attributed to preterm birth is more than $5.8 billion and was about 47% of medical care costs for all infant hospitalizations [5]. Despite the survival rates of preterm birth infants having increased these years, the disability of the infants has increased [6]. Prediction of preterm birth is important due to the enormous personal, economic and health implications of preterm birth. These predictions could provide reassurance for women who are less likely to give birth early while providing interventions for women who are likely to deliver prematurely.

Previous studies have reported that the risk of preterm birth was associated with age, race, smoking, economic status, and previous preterm birth [7, 8]. A single predictor may be weak in predicting preterm birth, while a better prediction can be obtained by combining a predictive model of multiple predictors [9]. Kim et al. conducted a systematic review summarizing current predictive models for predicting the risk of preterm birth [10]. The area under the receiver operating characteristic curve (AUC) for predicting preterm birth in these studies varied from 62 to 80%, and the effect of prediction was related to the number of predictors, populations, and the period of data (the first trimester, second trimester, etc.) [11–14]. Predicting the risk of preterm birth based on pre-pregnancy or first trimester data is more meaningful for the implementation of interventions. However, models with good predictive performance in this regard are rarely reported.

In this study, we aimed to establish a model to predict maternal preterm birth using baseline information that can predict preterm birth with the information readily available in clinical trials. Furthermore, external validation was conducted to assess the prediction ability of the model.

## Materials and methods

### Data source and participants

Data used in this case-control study were extracted from the National Vital Statistics System (NVSS) database between 2018 and 2019 [15]. The NVSS compiles the information from birth certificates and makes data files for each year, which is open access. The National Center for Health Statistics (NCHS) receives these electronic information files from the registration offices of all regions through the Vital Statistics Cooperative Program. The NVSS database has detailed data on each of the nearly 4 million births and 2.5 million deaths in the United States each year, including age, sex, race and ethnicity, and detailed geographic information. In addition, key indicators available in vital statistics such as infant mortality, access to antenatal care, maternal risk factors and pregnancy history, adolescent birth rates, etc. are included. Women with complete gestational age information were included in the study. Exclusion criteria were as follows: (1) missing data on the number of fetuses; (2) pregnant women and their spouses with incomplete basic information, including age, race, education. A total of 3,006,989 pregnant women in 2019 and 3,039,922 pregnant women in 2018 were extracted for analysis. The data used in this study from the open access NVSS database, and the relevant information of participants was anonymized and did not involve human intervention. Therefore, this study was granted exemption ethics approval by the Ethics Committee of Beijing Haidian Maternal and Child Healthcare Hospital.

### Data collection

Data of pregnant women were collected including age (< 35 years and ≥ 35 years), race (white, black, and others), education (high school or below, bachelor, and master or above), pre-pregnancy body mass index (BMI) (underweight, normal, overweight, and obesity), prenatal care (yes or no), pregnancy smoking (yes or no), pre-pregnancy diabetes (yes or no), gestation diabetes (yes or no), pre-pregnancy hypertension (yes or no), gestation hypertension (yes or no), hypertension eclampsia (yes or no), previous preterm birth (yes or no), infection (yes or no), plurality (yes or no), and preterm birth (yes or no). In addition, the age, race, and education of the pregnant spouse were also collected. The outcome of this study was preterm birth.

### Definition

#### Preterm birth

Preterm birth means births occurring before 37 completed weeks of gestation are preterm for purposes of classification consistent with the ICD-9 (International Classification of Diseases, Ninth Revision) and ICD-10 (International Classification of Diseases, Tenth Revision) definitions.

#### Education

Educational status was divided into three categories, high school or below, bachelor, and master or above. 8th grade or less, 9th through 12th grade with no diploma, high school graduate or GED completed, some college credit, but not a degree, associate degree (AA, AS) combined into high school and below. Master’s degree (MA, MS, MEng, MEd, MSW, MBA) and doctorate (PhD, EdD) or professional degree (MD, DDS, DVM, LLB, JD) merged into master’s degree and above.

#### Pre-pregnancy BMI

Pre-pregnancy BMI was calculated as: [mother’s pre-pregnancy weight (lb) / [mother’s height (in)]2] * 703 [16]. Pre-pregnancy BMI: underweight (< 18.5 kg/m2), normal (18.5–24.9 kg/m2), overweight (25–29.9 kg/m2), obesity (≥30 kg/m2). In this study Obesity I: 30.0–34.9, Obesity II: 35.0–39.9, Obesity III: ≥40.0 combined into obesity (≥30 kg/m2).

#### Prenatal care

Information on the timing and number of prenatal care visits was collected from the items “Date of first prenatal visit” (with a checkbox for “No prenatal care”) and “Total number of prenatal visits for this pregnancy.”

#### Smoking

All entries reporting packs of cigarettes are converted to the corresponding number of cigarettes (1 pack = 20 cigarette). If the mother reported smoking in any of the three trimesters of pregnancy she was classified as a smoker (smoked anytime during pregnancy).

#### Infections

Infections include gonorrhea, syphilis, chlamydia, hepatitis B and hepatitis C.

#### Plurality

Plurality was defined as twin, triplet, quadruplet, and quintuplet and higher-order births.

### Model development and validation

The 2019 data were randomly divided into the training set and testing set with a ratio of 1:1. Univariate and multivariate logistic regression analyses were conducted. Variables that were statistically significant in univariate analysis were included in multivariate analysis using backward stepwise regression. The odds ratio (OR) and 95% confidence interval (CI) were used to assess the effect of the variable on preterm birth. Characteristics of the pregnant women and their spouses (age, race, education) and variables that were statistically significant in the multivariate regression analysis were included in the prediction model. The 2018 data were utilized for external validation of the predictive model. The performance of the predictive model was assessed by area under the curve (AUC), accuracy, sensitivity, specificity, negative predictive value (NPV), and positive predictive value (PPV). A nomogram used to predict whether a pregnant woman had a preterm birth was drawn.

### Sample size and model power

The sample size was calculated using the PASS 15.0.5 software (NCSS, LLC, Kaysville, UT, USA). Calculations of sample size and model power were shown in Supplement Fig. 1. The proportions of the general pregnant population who experienced age ≥ 35 (17.2%), pre-pregnancy underweight (6.0%), pregnancy smoking (5.2%), gestation diabetes (11.0%), gestation hypertension (11.6%), and previous preterm birth (3.9%) were used to determine the sample size for this study [17–19]. Having experience of pre-pregnancy underweight was chosen as the independent variable since it obtained a higher sample size among the other calculated explanatory variables. The sample size calculation was as follows: the proportion of pregnancy women having experiences of pre-pregnancy underweight was 6.0%, a detectable odds ratio of 1.17, confidence level of 95% (α = 0.05, two-sided test), and power of 95%. The minimum total sample size was calculated to be 31,156. After adding a 5% non-response rate, the total calculated sample size was 32,714. The sample size of the training set and the testing set in this study were 1,503,495 and 1,503,494, respectively, which fully met the needs of the analysis. In addition, the power of the model was calculated to be 1.000 based on the AUC of the model in the testing set of 0.688 and the preterm birth rate of 10.8% (162,269/1,503,494).

### Statistical analysis

Categorical variables were described in numbers and percentages [n (%)] and the groups were compared using χ^2^ tests or Fisher’s exact tests. SAS 9.4 software (SAS Institute Inc., Cary, NC, USA) was used for analysis. R 4.02 software (Institute for Statistics and Mathematics, Vienna, Austria) was used to draw logistic prediction model nomogram. Python 3.7.3 software (Python Software Foundation, Delaware, USA) was utilized to calculate the AUC, accuracy, sensitivity, specificity, NPV, and PPV values. All statistical tests were used two-sided tests, and *P* < 0.05 was considered the difference to be statistically significant. Statistical power testing was performed using G*Power 3.1.9.7.

## Results

### Characteristics of participants

**Fig. 1:**
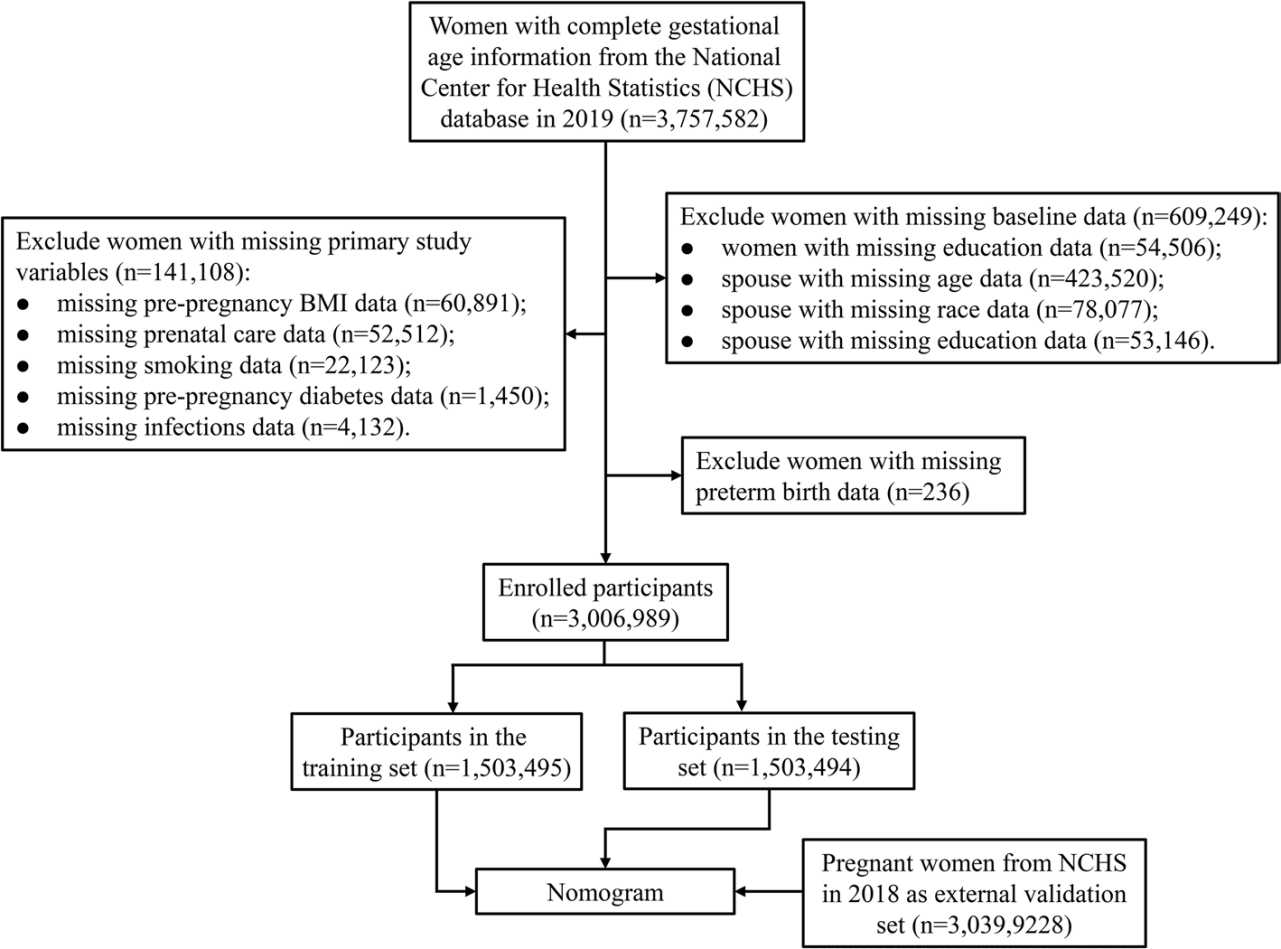
Flowchart for patient inclusion

Data of 3,757,582 pregnant women were extracted in 2019, and after excluding 750,593 pregnant women with incomplete information, a total of 3,006,989 women’s information were used for analysis (Fig. 1). Of these participants, 324,700 (10.80%) had preterm birth, 2,415,844 (80.34%) were < 35 years, 2,306,813 (76.72%) were whites, 431,342 (14.34%) were master or above, 1,254,721 (41.73%) had normal pre-pregnancy BMI, 28,389 (0.94%) had pre-pregnancy diabetes, 62,970 (2.09%) had pre-pregnancy hypertension, 230,989 (7.68%) had gestation hypertension, 7630 (0.25%) had hypertension eclampsia, 100,366 (3.34%) had previous preterm birth, and 94,853 (3.15%) had plurality. In addition, a total of 3,039,922 pregnant women in 2018 were included for external validation. Detailed characteristics of pregnant women in 2019 and 2018 were displayed in Table 1.

**Table 1:** Characteristics of all participants

Variables	2019 (***n*** = 3,006,989)	2018 (***n*** = 3,039,922)
Age (years), n (%)		
< 35	2,415,844 (80.34)	2,455,582 (80.78)
≥ 35	591,145 (19.66)	584,340 (19.22)
Race, n (%)		
White	2,306,813 (76.72)	2,342,911 (77.07)
Black	385,213 (12.81)	381,387 (12.55)
Other	314,963 (10.47)	315,624 (10.38)
Education, n (%)		
High school or below	1,868,219 (62.13)	1,902,319 (62.58)
Bachelor	707,428 (23.53)	708,539 (23.31)
Master or above	431,342 (14.34)	429,064 (14.11)
Age of spouse (years), n (%)		
< 35	1,125,350 (37.42)	1,149,463 (37.81)
≥ 35	1,881,639 (62.58)	1,890,459 (62.19)
Race of spouse, n (%)		
White	2,145,381 (71.35)	2,190,206 (72.05)
Black	437,487 (14.55)	432,525 (14.23)
Other	424,121 (14.10)	417,191 (13.72)
Education of spouse, n (%)		
High school or below	2,049,096 (68.14)	2,077,147 (68.33)
Bachelor	611,655 (20.34)	613,242 (20.17)
Master or above	346,238 (11.51)	349,533 (11.50)
Pre-pregnancy BMI (kg/m^2^), n (%)		
Underweight	86,346 (2.87)	91,667 (3.02)
Normal	1,254,721 (41.73)	1,302,458 (42.85)
Overweight	815,799 (27.13)	814,083 (26.78)
Obesity	850,123 (28.27)	831,714 (27.36)
Prenatal care, n (%)		
No	35,112 (1.17)	32,015 (1.05)
Yes	2,971,877 (98.83)	3,007,907 (98.95)
Pregnancy smoking, n (%)		
No	2,870,310 (95.45)	2,888,128 (95.01)
Yes	136,679 (4.55)	151,794 (4.99)
Pre-pregnancy diabetes, n (%)		
No	2,978,600 (99.06)	3,012,207 (99.09)
Yes	28,389 (0.94)	27,715 (0.91)
Gestation diabetes, n (%)		
No	2,791,240 (92.83)	2,828,904 (93.06)
Yes	215,749 (7.17)	211,018 (6.94)
Pre-pregnancy hypertension, n (%)		
No	2,944,019 (97.91)	2,980,984 (98.06)
Yes	62,970 (2.09)	58,938 (1.94)
Gestation hypertension, n (%)		
No	2,776,000 (92.32)	2,825,319 (92.94)
Yes	230,989 (7.68)	214,603 (7.06)
Hypertension eclampsia, n (%)		
No	2,999,359 (99.75)	3,032,983 (99.77)
Yes	7630 (0.25)	6939 (0.23)
Previous preterm birth, n (%)		
No	2,906,623 (96.66)	2,940,996 (96.75)
Yes	100,366 (3.34)	98,926 (3.25)
Previous cesareans, n (%)		
No	2,536,384 (84.35)	2,560,142 (84.22)
Yes	470,605 (15.65)	479,780 (15.78)
Infections, n (%)		
No	2,947,151 (98.01)	2,980,523 (98.05)
Yes	59,838 (1.99)	59,399 (1.95)
Plurality, n (%)		
No	2,912,136 (96.85)	2,942,193 (96.79)
Yes	94,853 (3.15)	97,729 (3.21)
Preterm birth, n (%)		
No	2,682,289 (89.20)	2,725,371 (89.65)
Yes	324,700 (10.80)	314,551 (10.35)

### Differences in women with and without preterm birth

Table 2 shows the differences in women with and without preterm birth. The results indicated that there were differences in age (*P* < 0.001), race (*P* < 0.001), education (*P* < 0.001), age of spouse (*P* < 0.001), race of spouse (*P* < 0.001), education of spouse (*P* < 0.001), pre-pregnancy BMI (*P* < 0.001), prenatal care (*P* < 0.001), pregnancy smoking (*P* < 0.001), pre-pregnancy diabetes (*P* < 0.001), gestation diabetes (*P* < 0.001), pre-pregnancy hypertension (*P* < 0.001), gestation hypertension (*P* < 0.001), hypertension eclampsia (*P* < 0.001), previous preterm birth (*P* < 0.001), previous cesareans (*P* < 0.001), infections (*P* < 0.001), and plurality (*P* < 0.001) between women with and without preterm birth.

**Table 2:** Differences in women with and without preterm birth in the training set

Variables	No preterm birth (***n*** = 1,341,064)	Preterm birth (***n*** = 162,431)	Statistic	***P***-value
Age (years), n (%)			χ^2^ = 1762.893	< 0.001
< 35	1,083,492 (80.79)	124,110 (76.41)		
≥ 35	257,572 (19.21)	38,321 (23.59)		
Race, n (%)			χ^2^ = 4117.058	< 0.001
White	1,036,752 (77.31)	116,955 (72.00)		
Black	163,447 (12.19)	28,920 (17.80)		
Other	140,865 (10.50)	16,556 (10.19)		
Education, n (%)			χ^2^ = 2515.445	< 0.001
High school or below	823,393 (61.40)	110,114 (67.79)		
Bachelor	321,473 (23.97)	32,480 (20.00)		
Master or above	196,198 (14.63)	19,837 (12.21)		
Age of spouse (years), n (%)			χ^2^ = 27.163	< 0.001
< 35	502,507 (37.47)	59,788 (36.81)		
≥ 35	838,557 (62.53)	102,643 (63.19)		
Race of spouse, n (%)			χ^2^ = 3841.278	< 0.001
White	965,494 (71.99)	107,583 (66.23)		
Black	186,516 (13.91)	31,739 (19.54)		
Other	189,054 (14.10)	23,109 (14.23)		
Education of spouse, n (%)			χ^2^ = 2421.738	< 0.001
High school or below	905,120 (67.49)	119,379 (73.50)		
Bachelor	278,139 (20.74)	27,937 (17.20)		
Master or above	157,805 (11.77)	15,115 (9.31)		
Pre-Pregnancy BMI (kg/m^2^), n (%)			χ^2^ = 1860.765	< 0.001
Underweight	37,969 (2.83)	5123 (3.15)		
Normal	566,124 (42.21)	61,369 (37.78)		
Overweight	364,452 (27.18)	43,258 (26.63)		
Obesity	372,519 (27.78)	52,681 (32.43)		
Prenatal care, n (%)			χ^2^ = 1499.057	< 0.001
No	14,127 (1.05)	3489 (2.15)		
Yes	1,326,937 (98.95)	158,942 (97.85)		
Pregnancy smoking, n (%)			χ^2^ = 1000.187	< 0.001
No	1,282,591 (95.64)	152,537 (93.91)		
Yes	58,473 (4.36)	9894 (6.09)		
Pre-pregnancy diabetes, n (%)			χ^2^ = 2960.522	< 0.001
No	1,330,375 (99.20)	158,888 (97.82)		
Yes	10,689 (0.80)	3543 (2.18)		
Gestation diabetes, n (%)			χ^2^ = 1822.489	< 0.001
No	1,248,946 (93.13)	146,570 (90.24)		
Yes	92,118 (6.87)	15,861 (9.76)		
Pre-pregnancy hypertension, n (%)			χ^2^ = 4859.660	< 0.001
No	1,316,914 (98.20)	155,257 (95.58)		
Yes	24,150 (1.80)	7174 (4.42)		
Gestation hypertension, n (%)			χ^2^ = 16,894.51	< 0.001
No	1,251,215 (93.30)	136,777 (84.21)		
Yes	89,849 (6.70)	25,654 (15.79)		
Hypertension eclampsia, n (%)			χ^2^ = 2871.288	< 0.001
No	1,338,663 (99.82)	160,985 (99.11)		
Yes	2401 (0.18)	1446 (0.89)		
Previous preterm birth, n (%)			χ^2^ = 15,999.98	< 0.001
No	1,304,996 (97.31)	148,372 (91.34)		
Yes	36,068 (2.69)	14,059 (8.66)		
Previous cesareans, n (%)			χ^2^ = 1441.546	< 0.001
No	1,136,372 (84.74)	131,751 (81.11)		
Yes	204,692 (15.26)	30,680 (18.89)		
Infections, n (%)			χ^2^ = 240.924	< 0.001
No	1,315,097 (98.06)	158,359 (97.49)		
Yes	25,967 (1.94)	4072 (2.51)		
Plurality, n (%)			χ^2^ = 107,024.1	< 0.001
No	1,320,388 (98.46)	135,475 (83.40)		
Yes	20,676 (1.54)	26,956 (16.60)		

### Factors associated with preterm birth

Table 3 demonstrates the univariate and multivariate analyses of factors associated with preterm birth. The univariate analysis found that age ≥ 35 years, black race of pregnant women and their spouses, pre-pregnancy overweight, underweight and obesity, pregnancy smoking, pre-pregnancy diabetes, gestation diabetes, pre-pregnancy hypertension, gestation hypertension, hypertension eclampsia, previous preterm birth, previous cesareans, infections, and plurality may be linked to a higher risk of preterm birth (all *P* < 0.05), and higher education level of pregnant women and their spouses and prenatal care may have a lower risk of preterm birth (*P* < 0.05). The multivariate analysis presented that higher education level of pregnant women [bachelor (OR = 0.82; 95%CI, 0.81–0.84); master or above (OR = 0.82; 95%CI, 0.81–0.83)] and their spouses [bachelor (OR = 0.86; 95%CI, 0.84–0.87); master or above (OR = 0.82; 95%CI, 0.80–0.84)], pre-pregnancy overweight (OR = 0.96; 95%CI, 0.95–0.98) and obesity (OR = 0.94; 95%CI, 0.93–0.96), and prenatal care (OR = 0.48; 95%CI, 0.47–0.50) were associated with a decreased risk of preterm birth, while age ≥ 35 years (OR = 1.27; 95%CI, 1.26–1.29), the black race of pregnant women (OR = 1.26; 95%CI, 1.23–1.29) and their spouses (OR = 1.15; 95%CI, 1.12–1.18), pre-pregnancy underweight (OR = 1.26; 95%CI, 1.22–1.30), pregnancy smoking (OR = 1.27; 95%CI, 1.24–1.30), pre-pregnancy diabetes (OR = 2.08; 95%CI, 1.99–2.16), gestation diabetes (OR = 1.27; 95%CI, 1.24–1.29), pre-pregnancy hypertension (OR = 2.22; 95%CI, 2.16–2.29), gestation hypertension (OR = 2.49; 95%CI, 2.45–2.53), hypertension eclampsia (OR = 4.12; 95%CI, 3.83–4.42), previous preterm birth (OR = 2.95; 95%CI, 2.88–3.01), previous cesareans (OR = 1.13; 95%CI, 1.11–1.14), infections (OR = 1.12; 95%CI, 1.08–1.16), and plurality (OR = 12.99; 95%CI, 12.73–13.24) were related to an increased risk of preterm birth.

**Table 3:** Univariate and multivariate analyses of factors associated with preterm birth

Variables	Univariate		Multivariate	
	OR (95%CI)	***P***	OR (95%CI)	***P***
Age				
< 35 years	Ref		Ref	
≥ 35 years	1.30 (1.28–1.32)	< 0.001	1.27 (1.26–1.29)	< 0.001
Race				
White	Ref		Ref	
Black	1.57 (1.55–1.59)	< 0.001	1.26 (1.23–1.29)	< 0.001
Others	1.04 (1.02–1.06)	< 0.001	1.05 (1.03–1.07)	< 0.001
Education				
High school or below	Ref		Ref	
Bachelor	0.76 (0.75–0.77)	< 0.001	0.82 (0.81–0.84)	< 0.001
Master or above	0.76 (0.74–0.77)	< 0.001	0.82 (0.81–0.83)	< 0.001
Age of spouse				
< 35 years	Ref		Ref	
≥ 35 years	1.03 (1.02–1.04)	< 0.001	0.98 (0.97–0.99)	< 0.001
Race of spouse				
White	Ref		Ref	
Black	1.53 (1.51–1.55)	< 0.001	1.15 (1.12–1.18)	< 0.001
Others	1.10 (1.08–1.11)	< 0.001	1.12 (1.10–1.14)	< 0.001
Education of spouse				
High school or below	Ref		Ref	
Bachelor	0.76 (0.75–0.77)	< 0.001	0.86 (0.84–0.87)	< 0.001
Master or above	0.73 (0.71–0.74)	< 0.001	0.82 (0.80–0.84)	< 0.001
Pre-pregnancy BMI				
Normal	Ref		Ref	
Underweight	1.24 (1.21–1.28)	< 0.001	1.26 (1.22–1.30)	< 0.001
Overweight	1.09 (1.08–1.11)	< 0.001	0.96 (0.95–0.98)	< 0.001
Obesity	1.30 (1.29–1.32)	< 0.001	0.94 (0.93–0.96)	< 0.001
Prenatal care				
No	Ref		Ref	
Yes	0.48 (0.47–0.50)	< 0.001	0.48 (0.47–0.50)	< 0.001
Pregnancy smoking				
No	Ref		Ref	
Yes	1.42 (1.39–1.46)	< 0.001	1.27 (1.24–1.30)	< 0.001
Pre-pregnancy diabetes				
No	Ref		Ref	
Yes	2.78 (2.67–2.88)	< 0.001	2.08 (1.99–2.16)	< 0.001
Gestation diabetes				
No	Ref		Ref	
Yes	1.47 (1.44–1.49)	< 0.001	1.27 (1.24–1.29)	< 0.001
Pre-pregnancy hypertension				
No	Ref		Ref	
Yes	2.52 (2.45–2.59)	< 0.001	2.22 (2.16–2.29)	< 0.001
Gestation hypertension				
No	Ref		Ref	
Yes	2.61 (2.57–2.65)	< 0.001	2.49 (2.45–2.53)	< 0.001
Hypertension eclampsia				
No	Ref		Ref	
Yes	5.01 (4.70–5.35)	< 0.001	4.12 (3.83–4.42)	< 0.001
Previous preterm birth				
No	Ref		Ref	
Yes	3.43 (3.36–3.50)	< 0.001	2.95 (2.88–3.01)	< 0.001
Previous cesareans				
No	Ref		Ref	
Yes	1.29 (1.28–1.31)	< 0.001	1.13 (1.11–1.14)	< 0.001
Infections				
No	Ref		Ref	
Yes	1.30 (1.26–1.35)	< 0.001	1.12 (1.08–1.16)	< 0.001
Plurality				
No	Ref		Ref	
Yes	12.71 (12.47–12.95)	< 0.001	12.99 (12.73–13.24)	< 0.001

### Model performance and validation

**Fig. 2:**
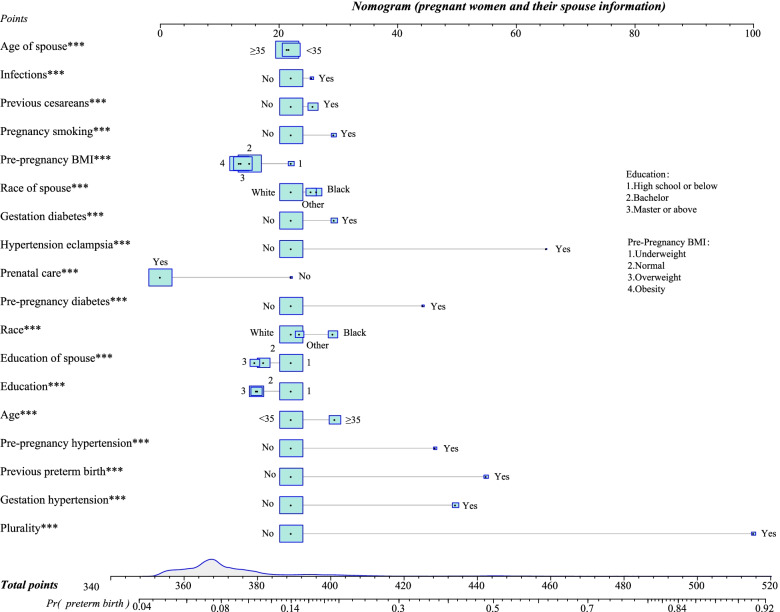
Nomogram for predicting the occurrence of preterm birth in pregnant women

Variables such as age, race, education of pregnant women and their spouses, pre-pregnancy BMI, prenatal care, pregnancy smoking, pre-pregnancy diabetes, gestation diabetes, pre-pregnancy hypertension, gestation hypertension, hypertension eclampsia, previous preterm birth, previous cesareans, infection, and plurality were included to develop a prediction model. Table 4 presents the model performance in the training set, testing set, and external validation set. According to the Yoden index, the cut-off point was 0.099. The AUC of the model in the training set, testing set, and external validation set was 0.689 (95%CI, 0.687–0.690), 0.688 (95%CI, 0.686–0.689), and 0.694 (95%CI, 0.693–0.695), respectively. The accuracy of the model in the training set, testing set, and external validation set was 0.763 (95%CI, 0.762–0.764), 0.762 (95%CI, 0.762–0.763), and 0.771 (95%CI, 0.770–0.771), respectively. Furthermore, the nomogram used to predict the occurrence of preterm birth in the pregnant woman was shown in Fig. 2.

**Table 4:** Performances of the model in the training set, testing set, and external validation set

Parameters	Training set	Testing set	Validation set
AUC (95%CI)	0.689 (0.687–0.690)	0.688 (0.686–0.689)	0.694 (0.693–0.695)
Accuracy (95%CI)	0.763 (0.762–0.764)	0.762 (0.762–0.763)	0.771 (0.770–0.771)
Specificity (95%CI)	0.796 (0.795–0.797)	0.796 (0.795–0.796)	0.803 (0.802–0.803)
Sensitivity (95%CI)	0.489 (0.487–0.492)	0.487 (0.484–0.489)	0.490 (0.488–0.492)
PPV (95%CI)	0.225 (0.224–0.226)	0.224 (0.222–0.225)	0.223 (0.222–0.224)
NPV (95%CI)	0.928 (0.927–0.928)	0.928 (0.927–0.928)	0.932 (0.931–0.932)

*AUC* Area under the curve, *PPV* Positive predictive value, *NPV* Negative predictive value, *CI* Confidence interval

## Discussion

In this study, we established a prediction model based on a large-sample database to predict preterm birth. Our results demonstrated that bachelor or above education level of pregnant women and their spouses, pre-pregnancy overweight and obesity, and prenatal care were linked to a reduced risk of preterm birth, while age ≥ 35 years, the black race of pregnant women and their spouses, pre-pregnancy underweight, pregnancy smoking, pre-pregnancy diabetes, gestation diabetes, pre-pregnancy hypertension, gestation hypertension, hypertension eclampsia, previous preterm birth, previous cesareans, infections, and plurality were associated with an increased risk of preterm birth. In the preterm birth prediction model constructed by these variables, the AUC of the model was 0.688 in the testing set. In addition, the model still performed well in the external validation set.

Black ethnicity and advanced maternal age may be indicators of preterm birth in some studies [2, 20]. In a meta-analysis examining racial differences among United States residents, black ethnicity had a higher rate of preterm birth than whites [20]. Our results showed that pregnant women aged ≥35 years and of the black race were associated with an increased risk of preterm birth. The relationship between pre-pregnancy BMI and preterm birth may influence by many factors [21, 22]. A large sample study indicated that pre-pregnancy obesity and preterm birth risk vary by age and race of pregnant women [21]. Our results found that pre-pregnancy overweight and obesity were linked to a decreased risk of preterm birth. This may be due to other confounders affecting our results. Furthermore, previous studies have reported that hypertension [23, 24] and diabetes [25] were associated with the risk of preterm birth. The risk of preterm birth increased with a plurality. Hiersch et al. found a higher rate of preterm birth in triplet pregnancies compared with twin pregnancies [26]. Smoking was also a risk factor which was a key necessary risk factor for fetal death or disability [27]. Our results showed that pregnancy smoking was related to an increased risk of preterm birth. In addition, our results found that prenatal care and higher education level were associated with a decreased risk of preterm birth. Previous studies have also shown that education was associated with a reduced risk of preterm birth in pregnant women, but the relationship is not linear [28, 29].

There were some studies focused on prediction models related to preterm birth, such as preterm birth prevention, cesarean delivery during the preterm period, or nulliparous women with a short cervix [11–14]. Many predictors were related to preterm birth such as general risk factors (maternal characteristics), pregnancy complication status (hypertension, diabetes), current pregnancy status, environmental complications, and medical intake [7]. During the pregnancy, the baseline information was much easier to collect. However, there were few studies to research the pregnant spouse’s information as the predictor. In our preterm birth prediction model, baseline information such as age, race, and education level of pregnant women and their spouses were included. The AUC of our prediction model in the testing set was 0.688. The prediction performance of our model may not be significantly improved compared to previous studies (0.688 vs. 0.63–0.74) [11–13]. However, our model was based on clinically readily available baseline information, which may increase the applicability of our model. In addition, compared with the baseline information, clinical indicators may also have an important role in predicting preterm birth. Some studies used clinical indicators as the risk factors to predict preterm birth [30–32], such as cervix length which was the strongest clinical predictor of preterm birth, ultrasound, and blood test, which were expensive or complicated to use in normal maternal people. Furthermore, a blood test can be selected as a biomarker to show whether the pregnant has inflammation or oxidative stress [33]. The general biological markers C-reactive protein (CRP), cytokines, 8-isoprostane, and 8-hydroxydeoxyguanosine could be tested through a blood test. Moreover, from a study, the measurement of nine cell-free RNA (cf-RNA) transcripts in maternal blood predicted gestational age with comparable accuracy to ultrasound [32]. However, in the low-resource area, the source of medical personnel and medical resource for blood tests were limited and unsupported to examine preterm birth. Therefore, convenient indicators or inexpensive access to get information is also necessary.

From the perspective of genetics, the neonatal father and mother were responsible for fetuses. Many parents were associated with maternal outcomes directly and indirectly [34]. Sparse researches were on parents’ demographic information, especially father’s baseline information. There were knowledge gaps between preterm birth and the father’s information. In Portuguese association had research on the mothers and fathers to support premature babies [35]. The research collected the father’s demographic information. However, it was not for the prediction model. In that cohort study, the author explored the consequences of premature delivery with information about mother’s and father’s low socioeconomic status. Thus, our study elucidates the prediction model with the father’s baseline information as a predicting factor.

This study used a lot of demographic information which can obtain easily from clinical data. Because there were many restrictions on pregnancy and medical resources, such as in rural areas or low-income families. Using this prediction model can filter some pregnant who may have a preterm birth. Then the doctor can provide some suggestions or give preventions for pregnant to prevent them from preterm birth. This prediction model can save humans, material resources, and time. There were some limitations in this study. Firstly, the external validation group was still from the United States. Preterm birth was a global public health problem. This external validation may ignore the different countries pregnant. Because in different countries, pregnancy may have various characteristics, this prediction model may have different effects to predict preterm birth. Secondly, although our data source was easy to obtain, the effect of prediction model performance was not accurate because there was no gold standard in this prediction model. If there were some other information such as blood test information, and mid-term cervical length value, the predictive ability may increase. Third, 98.8% of the included population had prenatal care, and the predictive effect of the model on preterm birth in the population without prenatal care may need further verification.

## Conclusion

A nomogram for predicting the risk of preterm birth in pregnant women was established. The prediction model had good performance in the testing set and external validation set. In addition, the relevant predictors of the prediction model are readily available clinically, and the nomogram may provide a simple tool for the prediction of preterm birth.

## Supplementary Information

**Additional file 1: Supplement Fig. 1.** Calculations of sample size and model power.

## Data Availability

The datasets generated and/or analyzed during the current study are available in the NVSS repository, https://www.cdc.gov/nchs/nvss/index.htm.

## Abbreviations

NVSS: National Vital Statistics System
NCHS: National Center for Health Statistics
BMI: Body mass index
OR: Odds ratio
CI: Confidence interval
AUC: Area under the curve
NPV: Negative predictive value
PPV: Positive predictive value
