# Supplementary material for: Maternal preterm birth prediction in the United States: a case-control database study
Source: BMC Pediatr. 2022 Sep 14;22:547. doi: 10.1186/s12887-022-03591-w (PMC9472432; doi:10.1186/s12887-022-03591-w)

**Supplement Figure 1**. Calculations of sample size and model power.

1. Sample size calculated by pre-pregnancy underweight variable:


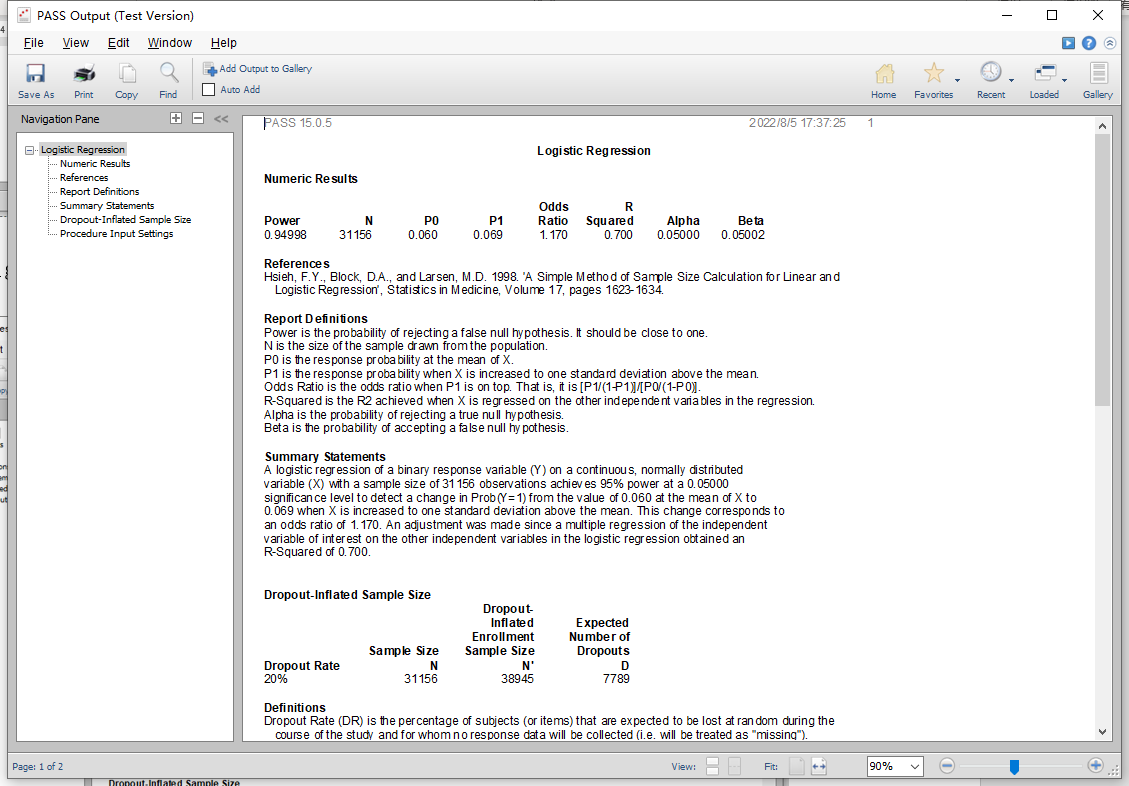


Note: N, number (sample size); P0, approximate proportion of the population exposed to this variable.

(2) Sample size calculated by age ≥35 variable:


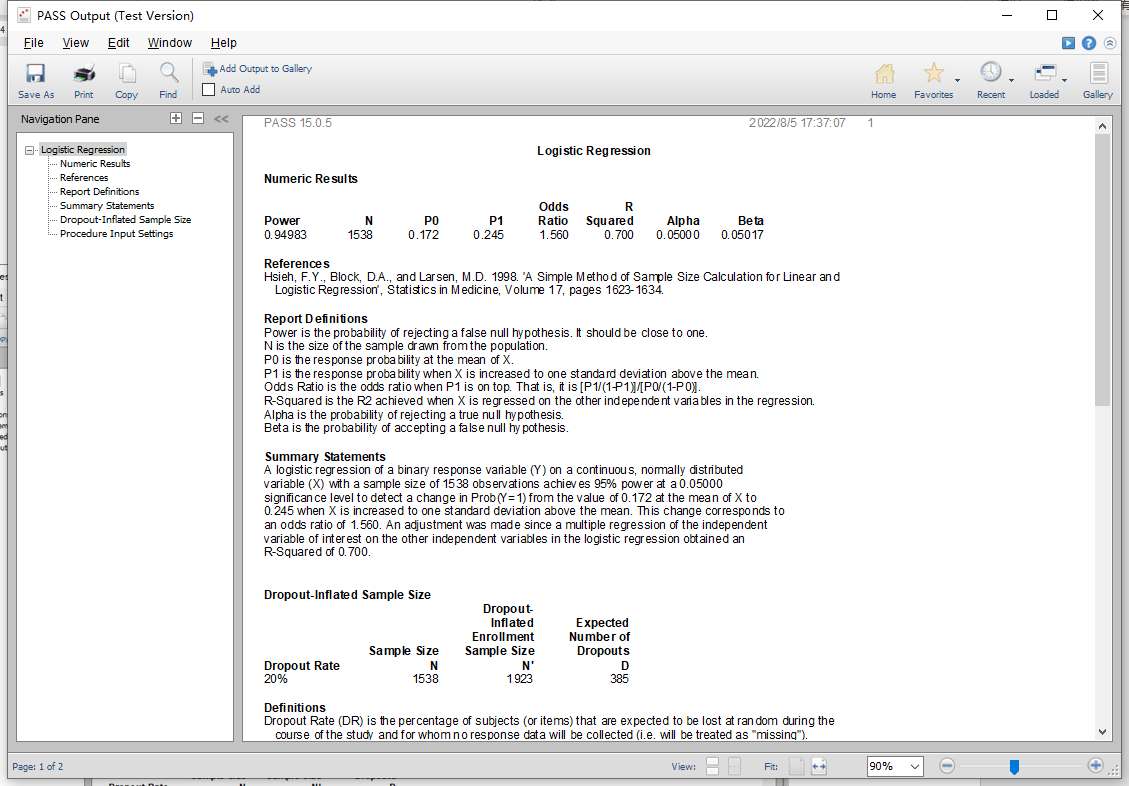


(3) Sample size calculated by pregnancy smoking variable:


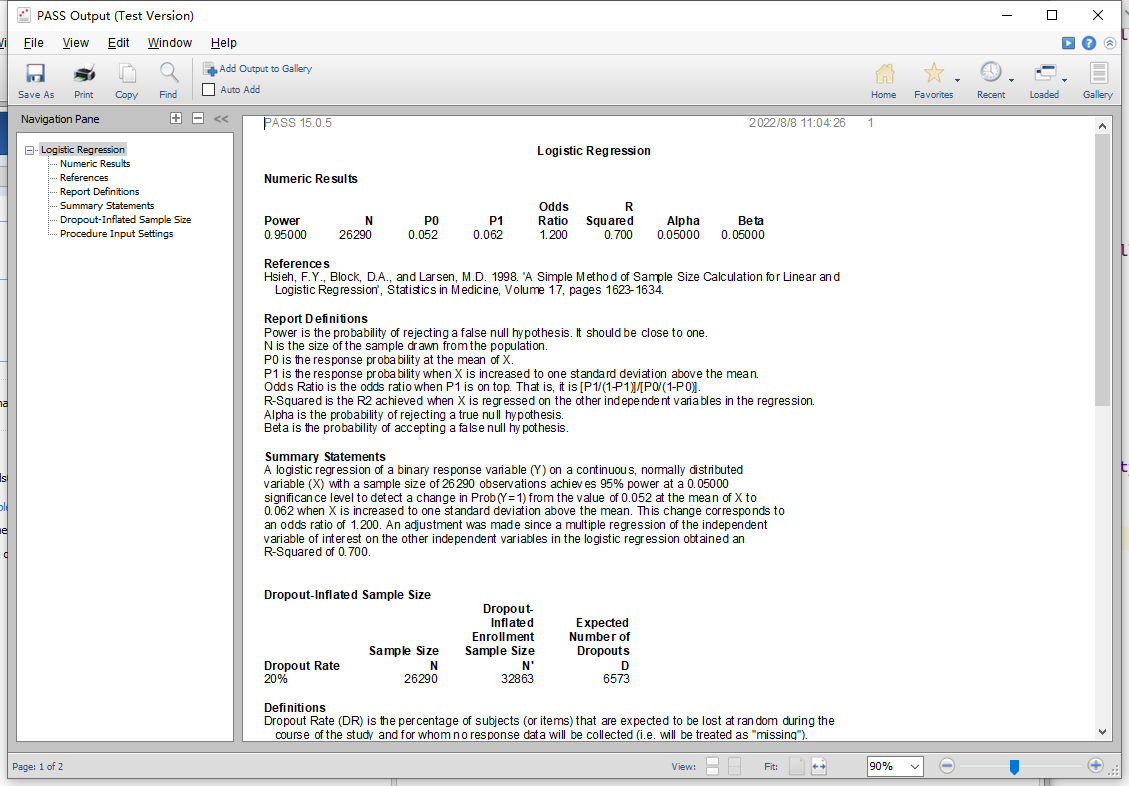


(4) Sample size calculated by gestation diabetes variable:


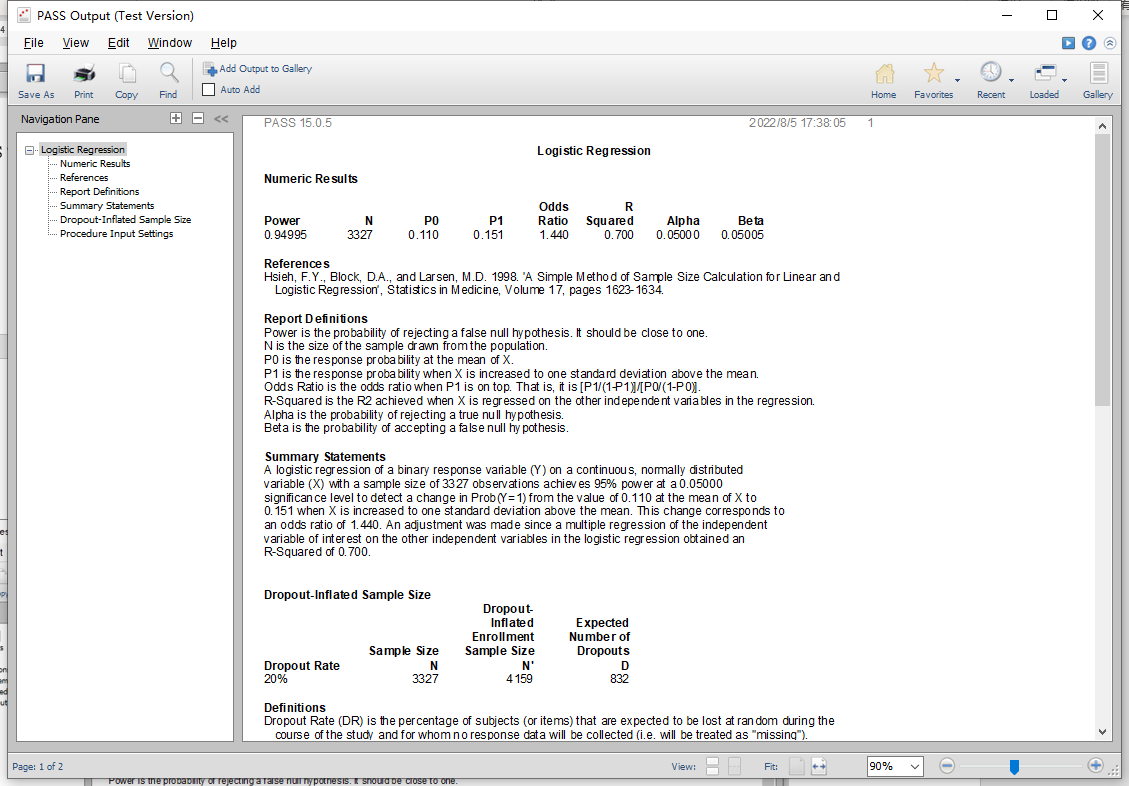


(5) Sample size calculated by gestation hypertension variable:


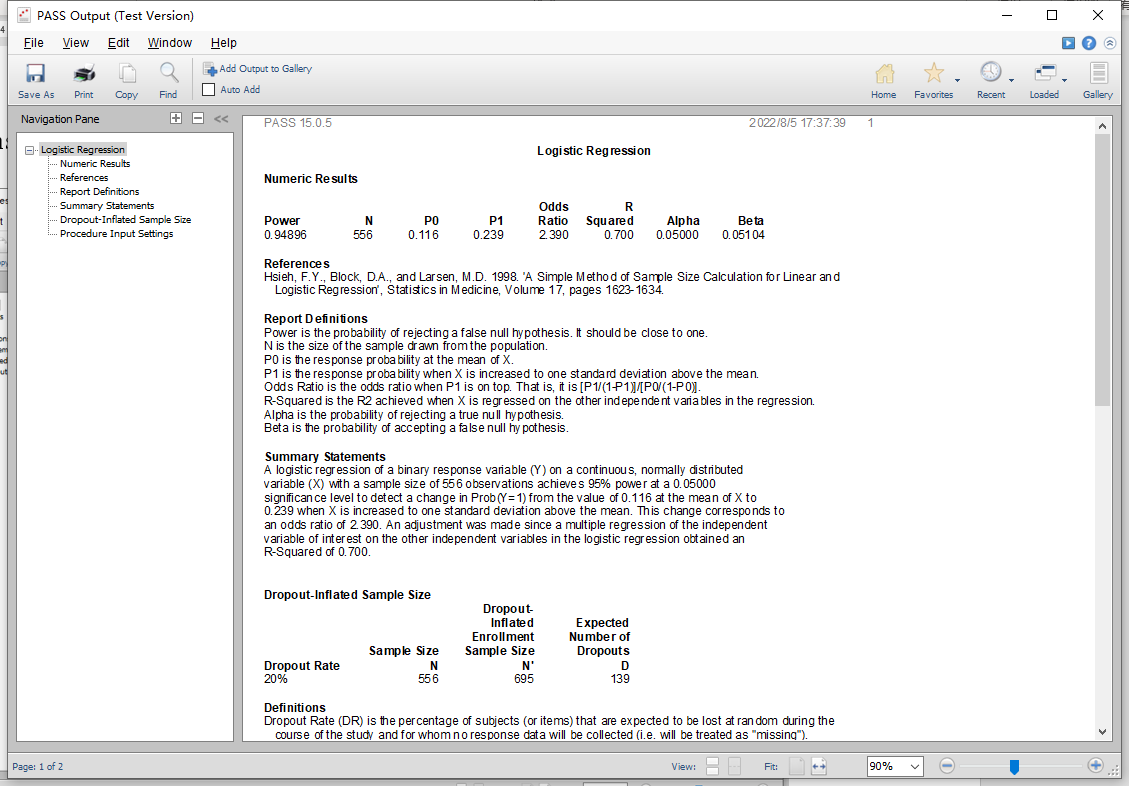


(6) Sample size calculated by previous preterm birth variable:


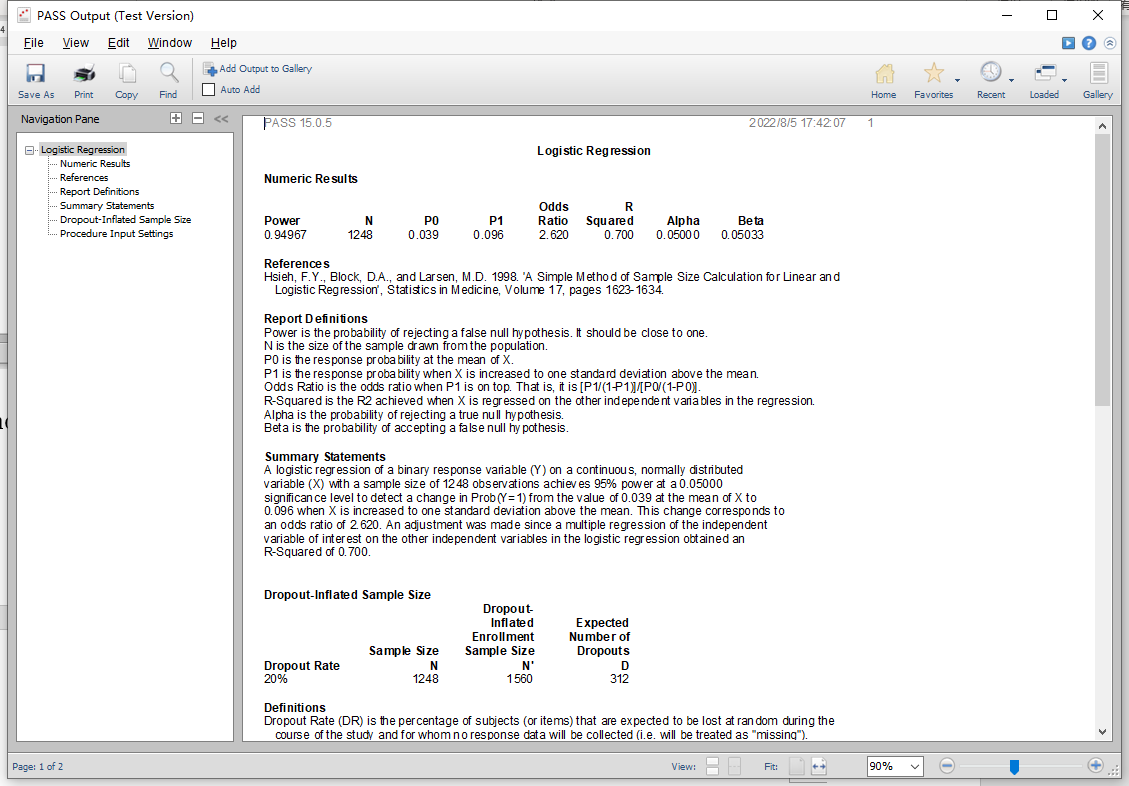


(7) Calculation of model power:


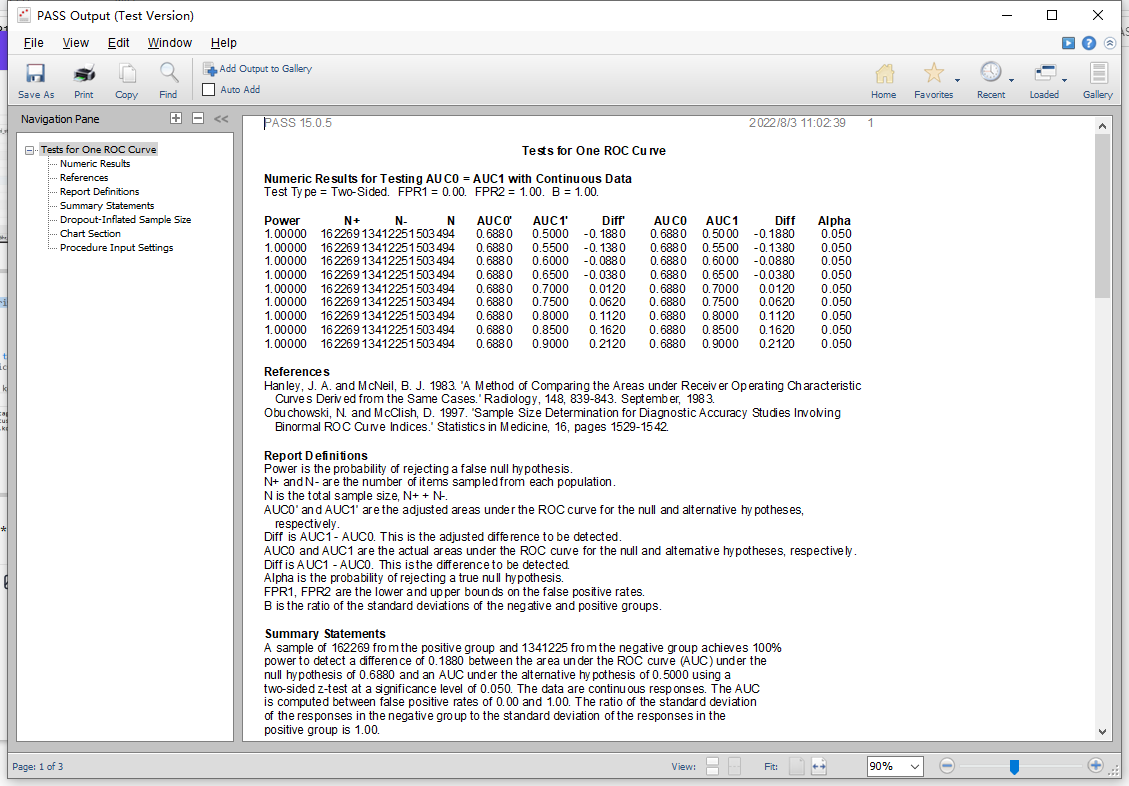

Supplement: Supplementary file 1 — Additional file 1: Supplement Fig. 1. Calculations of sample size and model power. [file 12887_2022_3591_MOESM1_ESM.docx]
